# Advances and bottlenecks in microbial hydrogen production

**DOI:** 10.1111/1751-7915.12790

**Published:** 2017-08-22

**Authors:** Alan J. Stephen, Sophie A. Archer, Rafael L. Orozco, Lynne E. Macaskie

**Affiliations:** ^1^ School ofChemical Engineering University of Birmingham Edgbaston Birmingham B15 2TT UK; ^2^ School of Biosciences University of Birmingham Edgbaston Birmingham B15 2TT UK

## Abstract

Biological production of hydrogen is poised to become a significant player in the future energy mix. This review highlights recent advances and bottlenecks in various approaches to biohydrogen processes, often in concert with management of organic wastes or waste CO_2_. Some key bottlenecks are highlighted in terms of the overall energy balance of the process and highlighting the need for economic and environmental life cycle analyses with regard also to socio‐economic and geographical issues.

## Introduction

Hydrogen provides a CO_2_‐free sustainable alternative to fossil fuels. A pioneering global initiative, the ‘Hydrogen Council’, comprising thirteen leading energy, transport and related industries, intends to increase investment in the hydrogen and fuel cell sectors (currently €1.4 Bn year^−1^) to stimulate hydrogen as a key part of the future energy mix via new policies and schemes (Anon, 2017).

Hydrogen is currently obtained mainly by steam reforming of hydrocarbons, releasing multiple greenhouse gas emissions (DOE, 2013). Hence, new H_2_ production methods are required such as biological production (bio‐H_2_; Dincer and Acar, 2015). H biotechnologies are maturing towards benchmarking against established clean energy from electrolysis of water, solar photovoltaics and wind farms. Biohydrogen can be made fermentatively from wastes, providing a simultaneous method of organic waste management (Chang *et al*., 2011). This short review highlights progress and bottlenecks of bio‐H_2_ towards a sustainable development goal to ensure access to affordable, reliable, sustainable and modern energy for all. Biohydrogen has been reviewed in comparison with other hydrogen production processes (Nikolaidis and Poullikkas, 2017).

Biohydrogen embraces any H_2_ production involving biological material (Mohan and Pandey, 2013). The energy source can be solar or can come from conversion of fixed carbon substrates (or both, in various combinations). An approach to CO_2_‐end of pipe treatment (e.g. from flue gas from fossil fuel combustion or carbon‐neutral fermentation of biomass) is to grow algae on waste CO_2_. Algal biohydrogen production is well‐described, but O_2_ from algal oxygenic photosynthesis inhibits the hydrogenase that makes H_2_. A key study (Kubas *et al*., 2017) will open the way to developing O_2_‐resistant hydrogenase. Emerging technology uses cyanobacteria (blue‐green algae) that make H_2_ via hydrogenase and also nitrogenase; their O_2_‐sensitivity is managed by temporal separation of photosynthetic O_2_ evolution and nitrogenase action, and by compartmentalization into microanaerobic heterocysts (Tiwari and Pandey, 2012). Despite a note that cyanobacterial biohydrogen is probably uneconomic (Singh *et al*., 2016), an environmental life cycle analysis (LCA) has shown for the first time that cyanobacterial bio‐H_2_ has the potential to be a competitor to desulfurized natural gas; the associated environmental impact of producing and extracting each gas, including use in a solid oxide fuel cell, was calculated and simulated respectively using the LCA software simapro (Archer *et al*., 2017). This research used published data from a raceway growth system (James *et al*., 2009). However, at latitudes above ~40°N, the generally low incident solar energy makes stand‐alone photobiological H_2_ systems seasonal and uneconomic without some form of process intensification. Boosting light delivery (e.g. LEDs, quantum dots) can be effective, but these may risk photopigment saturation and inhibition; this approach may be questionable economically and would be best addressed by a life cycle analysis. In sunny countries, light is plentiful, but in this case, ‘delivering cold’ is needed to extend crop product and food life; cooling is energy‐demanding and a global challenge (Strahan, 2017).

Another challenge is organic materials from agri‐food and municipal wastes, which must be managed to avoid landfilling which yields methane, a potent greenhouse gas. Current practices use anaerobic digestion (AD) with biogas – methane used for power. We review some options for combining waste treatments with bio‐H_2_ technology as possibly the best approach to tackling effectively these dual socio‐economic problems; stand‐alone biohydrogen is possibly uneconomic, but this awaits a life cycle analysis, currently in progress.

## Biohydrogen production from waste: fermentation strategies for sustainable ‘waste to hydrogen energy’

**Figure 1:**
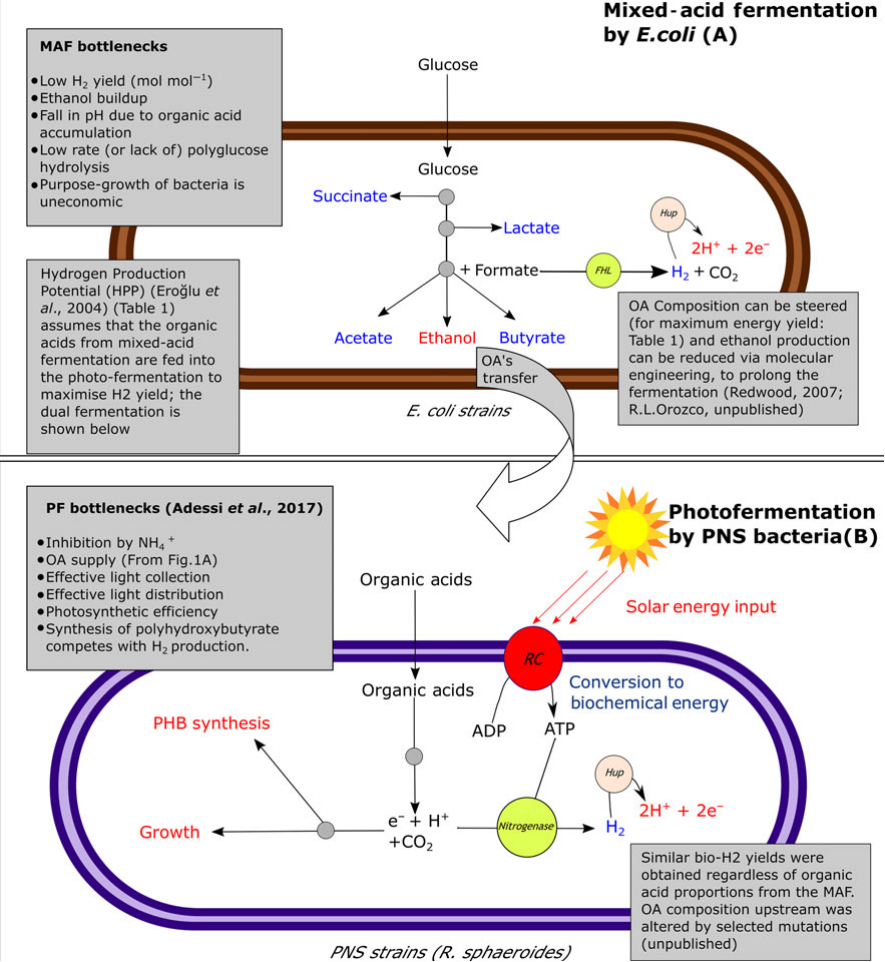
Mixed‐acid fermentation (MAF) of *E. coli* (A) and use of purple non‐sulfur bacteria (B) in photofermentation (PF) of organic acids (OAs) into H_2_. The organic acids are taken up by (e.g.) *R. sphaeroides,* and reducing power is generated as NADH (not shown). This reducing power can either be used for polyhydroxybutyrate synthesis or growth to maintain cellular redox or alternatively can be used for H_2_ production under light when growth is restricted by limitation of N or P source. Italicized bottlenecks are those overcome by use of the dual system (see text).

Fermentation is the disposal of excess metabolic reductant (NADH) onto organic compounds in the absence of alternative electron acceptors such as O_2_ and ${\text{NO}}_{3}^{-}$ (Guo *et al*., 2010). The mixed‐acid fermentation (‘dark fermentation’) pathway of the paradigm *Escherichia coli* (Fig. 1A) is simple, has high rates of H_2_ production but has limitations (Saratale *et al*., 2013; Fig. 1A inset). Hexose sugars can stoichiometrically deliver 12 mol H_2_ mol hexose^−1^. The mixed‐acid fermentation, while irreversible, is thermodynamically limited to 2–4 mol H_2_ mol hexose^−1^ (Hallenbeck, 2012). The ‘NADH pathway’ of some microorganisms (Hallenbeck, 2012, 2017) can deliver a higher H yield, but is reversible under a positive H_2_ partial pressure, which is required for with a downstream H fuel cell. Thermophilic bacteria have advantages but require input of heat energy. Hence, the focus has been mainly on mesophilic bacteria (Balachandar *et al*., 2013).

Most mixed‐acid fermentations follow a similar schematic: the cell forms reduced metabolic end‐products: organic acids (including toxic formate) and alcohol (Fig. 1A). Up to 2 mol H_2_ mol^−1^ hexose (Hallenbeck and Ghosh, 2009) is produced via the activity of formate hydrogen lyase (which splits formate to H_2_ + CO_2_), that is < 20% of the theoretical maximum H_2_. Sustained bio‐H_2_ production is limited by end‐product (ethanol) toxicity and acidification of the medium by accumulating organic acids (Redwood, 2007).

**Figure 2:**
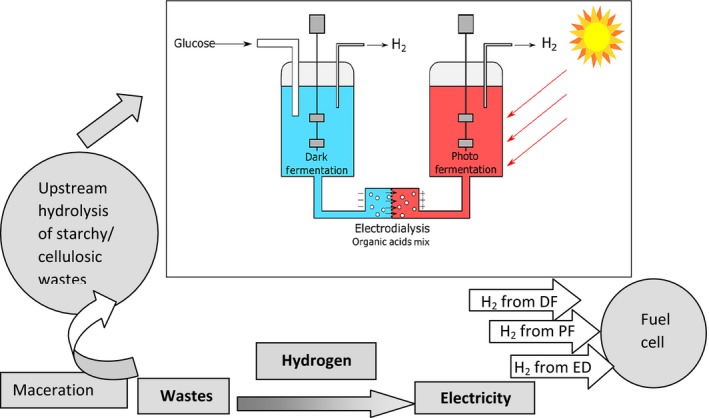
System for energy delivery from wastes via biohydrogen A fusion of chemical and biochemical engineering for conversion of waste into electricity via integrated biohydrogen technology. Electrodialysis (ED) separates the organic acid (OA) products from the mixed‐acid fermentation of (e.g.) *E. coli* (formate is converted to H_2_ + CO_2_ via formate hydrogen lyase). OAs pass from the dark fermentation medium to the photofermentation, typically being concentrated by ~eightfold via electrodialysis for dilution into the photofermentation vessel. Alcohol is not removed by ED; this would require a catalytic oxidation stage to give the corresponding organic acid; this has been achieved via using Au(0) nanoparticle catalyst made on *E. coli* cells (Deplanche *et al*., 2007). Two bio‐H_2_ streams are formed from the combined dark‐ and photofermentations, with a third H_2_ stream from electrolysis of water. The maximum H_2_ yield from the mixed‐acid fermentation is 2 mol sugar^−1^; hence, the dark fermentation can be viewed as a generator of OAs rather than as the primary H supply. A schematic of upstream waste conversion into sugar feed is shown (see text), and downstream use of hydrogen in a fuel cell for electricity production. Note that bio‐H_2_ is free of catalyst poisons, which extends fuel cell life. Not all wastes (e.g. sugary fruits, bakery products) require extensive upstream treatment. The main box is the biotechnology; the grey flow sheet is the chemical engineering required to realize the positive energy balance. Both are equally important.

The organic acids provide a means to overcome the thermodynamic limitation via their use in a coupled photofermentation reactor (Redwood *et al*., 2012a,b; Hallenbeck, 2013, 2017) via electrodialysis (Fig 2). If organic acid mixtures are fed to purple non‐sulfur bacteria (e.g. *Rhodobacter sphaeroides*), the off‐gas (typically > 90% H) is suitable for direct use in fuel cells (Nakada *et al*., 1999). This anoxygenic photofermentative H_2_ process (Fig. 1B) requires input of light energy (to help overcome the thermodynamic barrier in converting organic acids into H_2_ (Hallenbeck, 2013)). Nitrogen‐deficient conditions are essential; in purple non‐sulfur bacteria, H_2_ biogenesis is a side reaction of nitrogenase, which normally fixes N_2_ and is downregulated in the presence of fixed nitrogen. Utilizable organic acids also feed a competing pathway to make polyhydroxybutyrate which detracts from the H_2_ yield (Fig. 1B). Redwood *et al*. (2012a,b) incorporated an electrodialysis step to concentrate the organic acids (by ~eightfold) and link the mixed‐acid and photofermentation steps (Fig. 2). Electrodialysis separates anions (negatively charged organic acids in the dark fermentation medium), removing them and also preventing the transfer of inhibitory ${\text{NH}}_{4}^{+}$ into the photofermentation medium. This continuous dual fermentation process combines high H_2_ production rates and yield (Redwood *et al*., 2012b); the electrical energy demand of electrodialysis is counterbalanced, in part, by a third H_2_ stream from electrolysis of water.

Redwood (2007) calculated the break‐even current efficiency to quantify the role played by specific organic acids (Table 1). Butyrate is the most attractive organic acid for electrodialysis with the lowest break‐even current efficiency at 13% (Table 1). Butyrate is a neglected organic acid product from *E. coli* which can predominate under some conditions (Redwood, 2007; R.L. Orozco unpublished). Using this example (Figs 1 and 2), the energy balance for bio‐H_2_ (via fermentation of food waste) exceeded that from anaerobic digestion, wind and solar power, even without factoring in the additional electrochemically made H_2_. (Redwood *et al*., 2012b). Although ~half of the organic acid is available (anionic) at the pH of the fermentation (according to the p*K*_a_ values: Table 1), the electrodialysis chamber itself is alkaline due to OH^−^ release.

**Table 1:** Properties of organic acids relevant to their separation from spent medium by electrodialysis

Organic acid	Carbons	Valence	p*K*_a_	HPP mol^−1^	BCE (%)
Butyrate	4	1	4.81	10	13
Lactate	3	1	3.86	6	21.6
Formate	1	1	3.75	2	N/A
Acetate	2	1	4.76	7	32.5
Succinate	4	2	4.19, 5.57	7	27.1

The break‐even current efficiency (BCE: (energy expended/energy gained) × 100)) was calculated for individual organic acids. The lower the BCE, the less energy required to transport the organic acid. The electrical energy required for organic acid transport via electrodialysis relates to the number of charges and number of carbons; butyrate (4 carbons, 1 charge) is the most favourable and also has the highest proportion of charged butyrate (c.f. butyric acid) according to the *p*K_a_. HPP is hydrogen production potential of the dual system as defined by Eroğlu *et al*. (2004).

Two key findings are salient. First, the role of the dark fermentation is more important as a supply of organic acids into the photofermentation than for its bio‐H_2_ *per se*. Second, recent work (R.L. Orozco and A.J. Stephen, unpublished) showed that the H_2_ yield in the photofermentation was largely independent of the actual organic acid proportions in the feed from the mixed‐acid fermentation and was optimal at ~40 mM organic acids. Hence, any source of organic acids could be potentially used from a dual system or, indeed, in a stand‐alone photofermentation.

## Bacterial photofermentation

Purple non‐sulfur photosynthetic bacteria produce H_2_ from a variety of organic substrates including organic acids (Lazaro *et al*., 2012), sugars (Keskin and Hallenbeck, 2012) and industrial and agricultural effluents (Saratale *et al*., 2013), with high H_2_ yields from acetic, butyric and lactic acids (Hallenbeck, 2013). Bacteria used include *Rhodobacter sphaeroides* (Han *et al*., 2013)*, R. rubrum* (Zürrer and Bachofen, 1979)*, R. palustris* (Oh *et al*., 2004; Xiaobing, 2012) and *R. capsulatus* (Zhang *et al*., 2016); despite some differences, they all follow a similar general scheme (Fig. 1B), metabolizing organic acids to reduce NAD^+^ to the cellular reductant NADH (Oh *et al*., 2013). Excess reductant must be dissipated to reoxidize NADH and maintain cellular redox balance. This is achieved via cellular growth, channelling of carbon into cellular reserves (synthesis of polyhydroxybutyrate) or via H_2_ production under nitrogen‐deficient conditions, via nitrogenase, which produces H_2_ as an electron sink for excess reducing power (as with cyanobacteria: above). Nitrogenase normally fixes N_2_ into NH_3_ under light (to supply the large energy demand of N‐fixation, via ATP). Without N, the enzyme uses the reductant and ATP to produce H_2_ (2H^+^ + 2*e* ^−^ + 4 ATP ⇨ H_2_ + 4ADP + Pi). NADH is not a sufficiently strong reductant for this reaction; it is ‘upgraded’ to the stronger reductant ferredoxin via the input of energy, which is supplied by light through the action of the photosynthetic apparatus, via reverse electron transport. This apparatus also produces the ATP required for nitrogenase action (Hallenbeck, 2011). Various papers have studied the role of light (e.g. Uyar *et al*., 2007; Nath, 2009), showing that optimum light conversion efficiency occurs at light intensities much lower than light saturation points; e.g. Uyar *et al*. (2007) showed light saturation for *R. sphaeroides* at 270 W m^−2^ but similar substrate conversion efficiency could be achieved at light intensities as low 88 W m^−2^. Furthermore, optimum light intensities can be species specific; e.g. *R. sphaeroides* and *R. palustris* under similar conditions (Light intensity = 2500 Lux) had substrate conversion efficiencies of 60–70% and 47% respectively (Han *et al*., 2013; Oh *et al*., 2013).

Hallenbeck and Liu (2015) reviewed advances in the field, highlighting various approaches to improve substrate conversion efficiency (Table 2), while recent publications provide an up‐to‐date overview of recent developments for photobiological biohydrogen technologies (Adessi *et al*., 2017; Hallenbeck, 2017).

**Table 2:** Some approaches to increase photofermentation H productivity (Reviewed by Adessi *et al*., 2017)

Approach/Rationale	Outcomes/comments	References
‘Black box’ mathematical relationships between input and output streams Box‐behnken statistical design/methods	Permits multivariable analysis: measures cause and effect; hence can be empirical SCE (glycerol) > doubled (*R.palustris*)	Abo‐Hashesh *et al*. (2013), Show and Lee (2013) and Ghosh *et al*. (2012a,b,c)
Modelling metabolic fluxes	Guided interventions: success using lactate but not malate or acetate	Golomysova *et al*. (2010) and Hädicke *et al*. (2011)
Deletion of polyhydroxybutyrate synthesis pathway	Increased H_2_ yield (by 1.5‐fold c.f. wild type)	Kim *et al*. (2011)
Reducing pigment concentration	Allows greater light penetration^a^	Ma *et al*. (2012)
Use of quantum dots to ‘upgrade’ light	Doubled photosynthetic efficiency	M.D. Redwood, unpublished^b^

SCE, substrate conversion efficiency.

a 27% increase in H_2_ yield was obtained.

b Collaborative study with Photon Science Institute, University of Manchester: M.D. Redwood, L.E. Macaskie and D.J. Binks, unpublished work. But note: current commercial quantum dots would be grossly uneconomic at scale.

## Towards an economically competitive biohydrogen process from waste

Table 3 summarizes various options for a biohydrogen process. In the UK, food wastes at scale are generally centralized and ‘committed’ by agreements into anaerobic digestion and a ‘bolt‐on’ addition into existing anaerobic digestion and combined heat and power (CHP) processes is one option as there is insufficient waste available for a realistic stand‐alone bio‐H_2_ process (unpublished survey; Sustainable Resource Solutions Ltd). Agricultural wastes are currently unattractive due to high energy demands of comminution/maceration and upstream hydrolysis. A survey of wastes has indicated that vinasse (from bioethanol production) and in‐process streams from UK Utility companies contain sufficient organic acids to warrant trialling for data into a full life cycle analysis.

**Table 3:** Options for delivery of bio‐H_2_ into power, all via electro‐photofermentation (Figs 1 and 2; M.D. Redwood, R.L.Orozco and L.E. Macaskie, unpublished work)^a^

Feedstock (upstream)	Power (downstream)	Comments
Fermentation of food wastes	Fuel cell electricity^b^ or combined heat and power^c^	Food wastes (FW) required (tonnages). Anaerobic digestion (AD) has monopoly on FW. Bio‐H_2_ can power a fuel cell directly.
Fermentation of cellulosic wastes	Fuel cell electricity or CHP	Comminution/maceration energy demand adversely affects overall energy balance^e^. Upstream hydrolysis is required.
OAs obtained from anaerobic digestion (AD)	‘Hythane’: mix of CH_4_ (AD) + bio‐H_2_; CHP	AD interrupted at acetogenesis stage; organic acids diverted into a bolt‐on photofermentation. Overall AD residence time is reduced. This increases process complexity but gives a higher energy output. Gas is compatible with current infrastructure. Scenario 1: 20% more power^d^. Scenario 2: 70% more power^d^
OAs used directly from wastes (e.g. wastewaters) or CHP	Fuel cell electricity	Organic acid waste streams (tonnage scale) are (e.g.) vinasse (from bioethanol production) and municipal wastewater treatment plants (see text).

a Calculations were made independently of incentivization schemes as these tend to be ephemeral and skew the longer term picture. Likewise, increasing/decreasing feed‐in tariffs would complicate economic assessments.

b Fuel cell technology is still emergent at large scale, and FCs fail prematurely (see Rabis *et al*., 2012).

c Combined heat and power (CHP: well‐established technology). In this scenario, the methane stream from anaerobic digestion can be supplemented with photofermentatively derived H_2_ to make ‘hythane’ for CHP.

d Scenario 1: diversion of 10% of the organic acids into photofermentation and use of hythane in CHP. Scenario 2: diversion of 80% of the organic acids into photofermentation and use of AD‐methane in CHP plus use of the photofermentation H_2_ in a fuel cell would give 70% more power (R.L. Orozco, unpublished). The proportion of flow diverted from the acetogenesis step of anaerobic digestion (via electroseparation) could be simply ramped in response to incident light intensity to feed the photofermentation; at night the flow would pass to the methanogenic reactor as normal. By combining the two processes, the residence time in the system would also be reduced as compared to traditional anaerobic digestion due to reduced flow entering the methanogenesis reactor daily.

e Using *Miscanthus* as an example, the energy demand of comminution to 4 mm particles is 184 kJ kg dry matter^−1^; energy from H is 10 kJ l^−1^ (at 1 atm and 125°C); that from the dark fermentation was only 110 kJ kg cellulose; hydrolysate; hence the PF (~4 times the H_2_ as the dark fermentation) is key to a positive energy balance from complex substrates.

The organic acid content of a typical vinasse waste is > 40 g l^−1^ (Ryznar‐Luty *et al*., 2008; Esapaňa‐Gamboa *et al*., 2012); the high concentration of betaine (trimethylglycine, a zwitterionic osmoprotectant; 20 g l^−1^) is not potentially problematic because at the low pH of vinasse (pH 3–4), it would be protonated (i.e. inaccessible to the anion transfer in electroseparation). Moreover, betaine was reported to stimulate nitrogenase activity, but it was not used as a nitrogen source (Igeňo *et al*., 1995).

**Figure 3:**
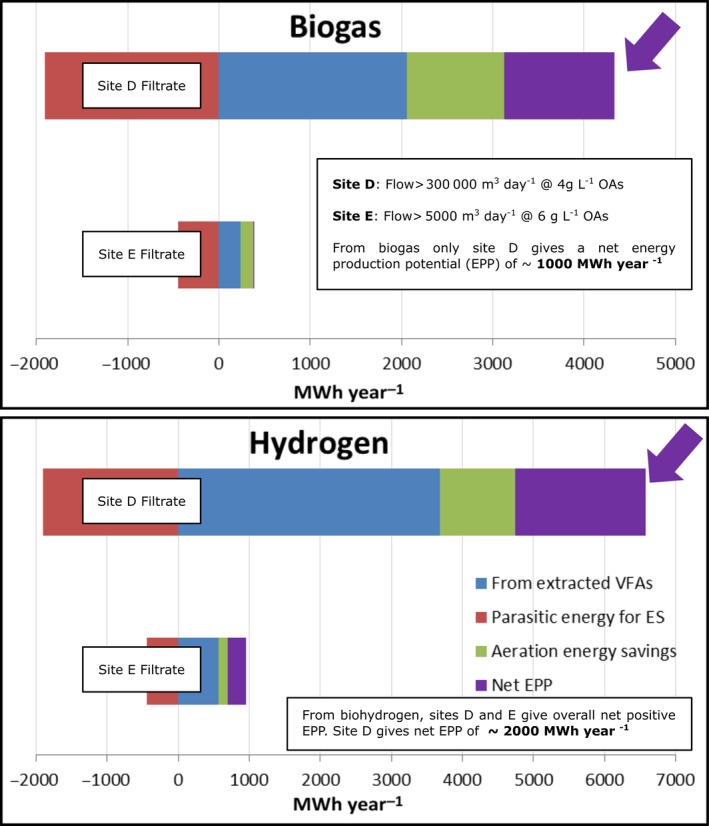
Energy production potential (EPP) from use of real wastewater organic acids in a stand‐alone photofermentation (real test data using *R. sphaeroides:* R.L.Orozco, I. Mikheenko and L.E. Macaskie, unpublished). As an organic acids liquid stream is used directly, the upstream dark fermentation is not required, and there is no sacrificial energy demand for maceration.

Selected UK utility company wastewaters were trialled as potential targets for hydrogen bioenergy following filtration to remove debris but with no other modifications (Fig. 3). The energy production potential from biohydrogen via photofermentation was twice that from biogas (Fig. 3). Hence, H_2_ energy from organic acid wastes is a viable option for energy production by heavily populated, industrialized countries but may be limited seasonally by available natural sunlight. Stand‐alone photobiological hydrogen production has major potential in solar‐rich countries with the option to also treat wastes in areas of high population density. An environmental life cycle analysis has been developed for cyanobacterial bio‐H_2_ (Archer *et al*., 2017). The next step is to apply a similar LCA for various options with respect to geographical location, other socio‐economic factors and the global increase in demand for cooling to safeguard food supplies for expanding populations.

## Conflict of Interest

None declared.
